# Social Relationships and Social Engagement Among African American Adults With a History of Incarceration

**DOI:** 10.1093/geroni/igab046.1179

**Published:** 2021-12-17

**Authors:** Rodlescia Sneed, Bridget Farmer, Jennifer Johnson

**Affiliations:** Michigan State University, Flint, Michigan, United States

## Abstract

Strong social relationships and social engagement are crucial for both successful aging and successful community re-entry after incarceration. Here, we utilized a mixed methods approach to understand the impact of incarceration on social relationships and social engagement among formerly incarcerated community-dwelling African-American adults aged >50. Participants in the 2012 or 2014 waves of the Health and Retirement Study answered questions regarding prior incarceration, social relationships, and participation in social activities. Additionally, we utilized key informant interviews to further explore how incarceration might impact relationships and social engagement. This presentation will describe quantitative associations between prior incarceration and social relationship structure & function. Further, we will use our qualitative interview data to further explore possible explanations for our findings. Finally, we will describe how MCUAAAR Scientist/Faculty interactions facilitated this work.

